# ASO Author Reflections: Radiopathomics Strategy of Combing Multi-scale Tumor Information on Pretreatment to Predict the Pathologic Response to Neoadjuvant Therapy

**DOI:** 10.1245/s10434-020-08700-6

**Published:** 2020-06-03

**Authors:** Jie Tian, Xinjuan Fan, Ruihua Xu, Ying-Shi Sun, Guanyu Yang

**Affiliations:** 1 CAS Key Laboratory of Molecular Imaging, Institute of Automation, Beijing, China; 2 School of Artificial Intelligence, University of Chinese Academy of Sciences, Beijing, China; 3 Beijing Advanced Innovation Center for Big Data-Based Precision Medicine, School of Medicine, Beihang University, Beijing, China; 4 Engineering Research Center of Molecular and Neuro Imaging of Ministry of Education, School of Life Science and Technology, Xidian University, Xi’an, China; 5 Department of Pathology, the Sixth Affiliated Hospital, Sun Yat-sen University, Guangzhou, China; 6 State Key Laboratory of Oncology in South China, Collaborative Innovation Center for Cancer Medicine, Sun Yat-sen University Cancer Center, Guangzhou, China; 7 Key Laboratory of Carcinogenesis and Translational Research (Ministry of Education/Beijing), Department of Radiology, Peking University Cancer Hospital and Institute, Beijing, China; 8 School of Computer Science and Engineering, Southeast University, Nanjing, China

## Past

The standard treatment for locally advanced rectal cancer (LARC) includes neoadjuvant chemoradiotherapy (nCRT) followed by total mesorectal excision (TME) and adjuvant chemotherapy.^1^ After nCRT, 15–27% of patients with LARC achieve a pathologic complete response (pCR) and usually have perfect long-term outcomes. These patients prefer to avoid surgery and preserve organs with a strategy such as “watch and wait” management.^2^ Additionally, for more than 50% of patients who cannot reach a good response (GR),^3^ treatment optimization according to different pathologic responses is essential to balance the benefits of nCRT against toxicity.^4^ Due to the advantages of radiomics for quantitative analysis of tumors,^5^ radiomics has demonstrated the potential of magnetic resonance imaging (MRI) in preoperative accurate evaluation of pCR^6^ or no response^7^ in previous studies. Furthermore, pretreatment multi-parameter magnetic resonance imaging (mp-MRI)-based radiomics was attempted to predict non-response to nCRT.^8^ However, to date, no nomogram has been established or acknowledged for predicting discrepancies in the response before nCRT.

## Present

In this study,^9^ 981 consecutive patients with evaluation of response according to tumor regression grade (TRG) who received nCRT (primary cohort and external validation cohorts 1–3) were retrospectively recruited from four Chinese hospitals. Each recruited patient had received both a pretreatment multi-parametric magnetic resonance imaging (mp-MRI) and a whole-slide image (WSI) of biopsy specimens. Quantitative image features were extracted from the mp-MRI and WSI. These features then were used for radiopathomics signature (RPS) construction powered by an artificial intelligence model. The predicted signature from the radiopathomics model yielded an overall accuracy (ACC) of 79.66–87.66% in the validation cohorts (VCs). The areas under the curve (AUCs) of RPS at specific response grades were 0.98 (TRG0), 0.93 (≤ TRG1), and 0.84 (≤ TRG2). The RPS at each grade of pathologic response showed significant improvement over signature construction without combining multi-scale tumor information (*P* < 0.01). The authors’ proposed radiopathomics strategy and signature escaped the limitation of using only medical imaging to depict the whole tumor and decreased the potential risk of overlooking tumor heterogeneity by adding micro-scale pathologic information.

## Future

This study provided evidence that the radiopathomics strategy of combing the images of both radiology and pathology is a potential strategy for predicting the variation in pathologic response before nCRT. More quantitative and multi-scale tumor information powered by the information fusion method was able to improve the heterogeneous description of tumors and enhance the performance of the model for restaging patients. As such, future studies should focus on the integration of gene and protein information to construct a more comprehensive tumor prediction model from the macro radiological information of tumor to the micro pathological information of tumor. The biologic interpretation between radiomic and pathomic features also should be central to future studies.
